# Fe‐containing metal–organic framework with D‐penicillamine for cancer‐specific hydrogen peroxide generation and enhanced chemodynamic therapy

**DOI:** 10.1002/btm2.10477

**Published:** 2023-02-01

**Authors:** Han Bi Ji, Cho Rim Kim, Chang Hee Min, Jae Hoon Han, Se‐Na Kim, Cheol Lee, Young Bin Choy

**Affiliations:** ^1^ Interdisciplinary Program in Bioengineering College of Engineering, Seoul National University Seoul Republic of Korea; ^2^ Institute of Medical & Biological Engineering, Medical Research Center, Seoul National University Seoul Republic of Korea; ^3^ Department of Pathology Seoul National University College of Medicine Seoul Republic of Korea; ^4^ Department of Biomedical Engineering Seoul National University College of Medicine Seoul Republic of Korea

**Keywords:** cancer specific, chemodynamic therapy, drug delivery, hydrogen peroxide, metal–organic framework

## Abstract

Chemodynamic therapy (CDT) is based on the production of cytotoxic reactive oxygen species, such as hydroxyl radicals (^•^OH). Thus, CDT can be advantageous when it is cancer‐specific, in terms of efficacy and safety. Therefore, we propose NH_2_‐MIL‐101(Fe), a Fe‐containing metal–organic framework (MOF), as a carrier of Cu (copper)‐chelating agent, d‐penicillamine (d‐pen; i.e., the NH_2_‐MIL‐101(Fe)/d‐pen), as well as a catalyst with Fe‐metal clusters for Fenton reaction. NH_2_‐MIL‐101(Fe)/d‐pen in the form of nanoparticles was efficiently taken into cancer cells and released d‐pen in a sustained manner. The released d‐pen chelated Cu that is highly expressed in cancer environments and this produces extra H_2_O_2_, which is then decomposed by Fe in NH_2_‐MIL‐101(Fe) to generate ^•^OH. Therefore, the cytotoxicity of NH_2_‐MIL‐101(Fe)/d‐pen was observed in cancer cells, not in normal cells. We also suggest a formulation of NH_2_‐MIL‐101(Fe)/d‐pen combined with NH_2_‐MIL‐101(Fe) loaded with the chemotherapeutic drug, irinotecan (CPT‐11; NH_2_‐MIL‐101(Fe)/CPT‐11). When intratumorally injected into tumor‐bearing mice in vivo, this combined formulation exhibited the most prominent anticancer effects among all tested formulations, owing to the synergistic effect of CDT and chemotherapy.

## 1 INTRODUCTION

Reactive oxygen species (ROS) are small, highly reactive molecules formed due to incomplete oxygen reduction.^1^ Recently, numerous studies have indicated that high levels of ROS can cause oxidative stress, thus damaging the cellular components of lipids, enzymes, proteins, and DNAs/RNAs, and inducing cell apoptosis.^2^, ^3^ Thus, this is being adopted as a potential approach in cancer therapy, as excessive ROS can increase oxidative damages, thus destroying cancer cells and inhibiting their proliferation.^4^ Therefore, a ROS‐mediated therapy called chemodynamic therapy (CDT) is being widely studied as a potent cancer treatment.^5^, ^6^

CDT is generally based on Fenton and Fenton‐like reactions, in which highly cytotoxic hydroxyl radicals are generated from hydrogen peroxide in the presence of Fe ions as catalysts.^7^, ^8^ Due to their strong reactivity with biomolecules, hydroxyl radicals are known to be more damaging to cancer cells than any other type of ROS.^9^ Therefore, there has been growing interest in the development of therapeutic formulations that can produce high levels of hydroxyl radicals via the Fenton reaction. Previous studies delivered Fe‐containing particles, such as Fe_3_O_4_, α‐Fe_2_O_3_, MnFe_2_O_4_, FePt, and FeO_x_‐mesoporous silica nanoparticles (MSNs), to cancer environments, as key catalysts, which reacted with endogenous H_2_O_2_ in cancer cells to generate hydroxyl radicals.^7^, ^10^ However, these particles were not stable in aqueous media, and their reaction was not efficient, most of which occurred only on the particle surfaces.^11^, ^12^, ^13^ Moreover, even in the presence of Fe ions, endogenous H_2_O_2_, albeit higher in cancer environments, is not yet sufficient to generate therapeutic levels of ROS that effectively kill cancer cells, resulting in low treatment efficacy.^14^, ^15^

A variety of strategies have been proposed to provide more H_2_O_2_ to cancer cells. For example, a bolus H_2_O_2_ solution was directly injected into the target site; however, it was not retained but rapidly cleared.^16^, ^17^ As an alternative approach, biocatalysts were administered to catalyze the oxidation of endogenous molecules to induce exogenous H_2_O_2_. For example, glucose oxidase (GOx) was utilized to elevate H_2_O_2_ concentration through a glucose metabolic reaction.^18^ In another study, NADPH oxidase (NOX) and superoxide dismutase (SOD) were utilized together. NOX catalyzed the conversion of endogenous oxygen into O_2_^•−^, which in turn is converted into H_2_O_2_ via an SOD‐catalyzed reaction.^19^ Although showing the improved efficacy of CDT with extra H_2_O_2_ production, the enzymes of natural proteins still pose challenges, such as immunogenicity, low stability, and high cost.^20^ Furthermore, endogenous molecules, such as glucose and oxygen, are ubiquitous; thus, systemic generation of H_2_O_2_ may still occur to induce side effects.^21^, ^22^

**SCHEME 1:**
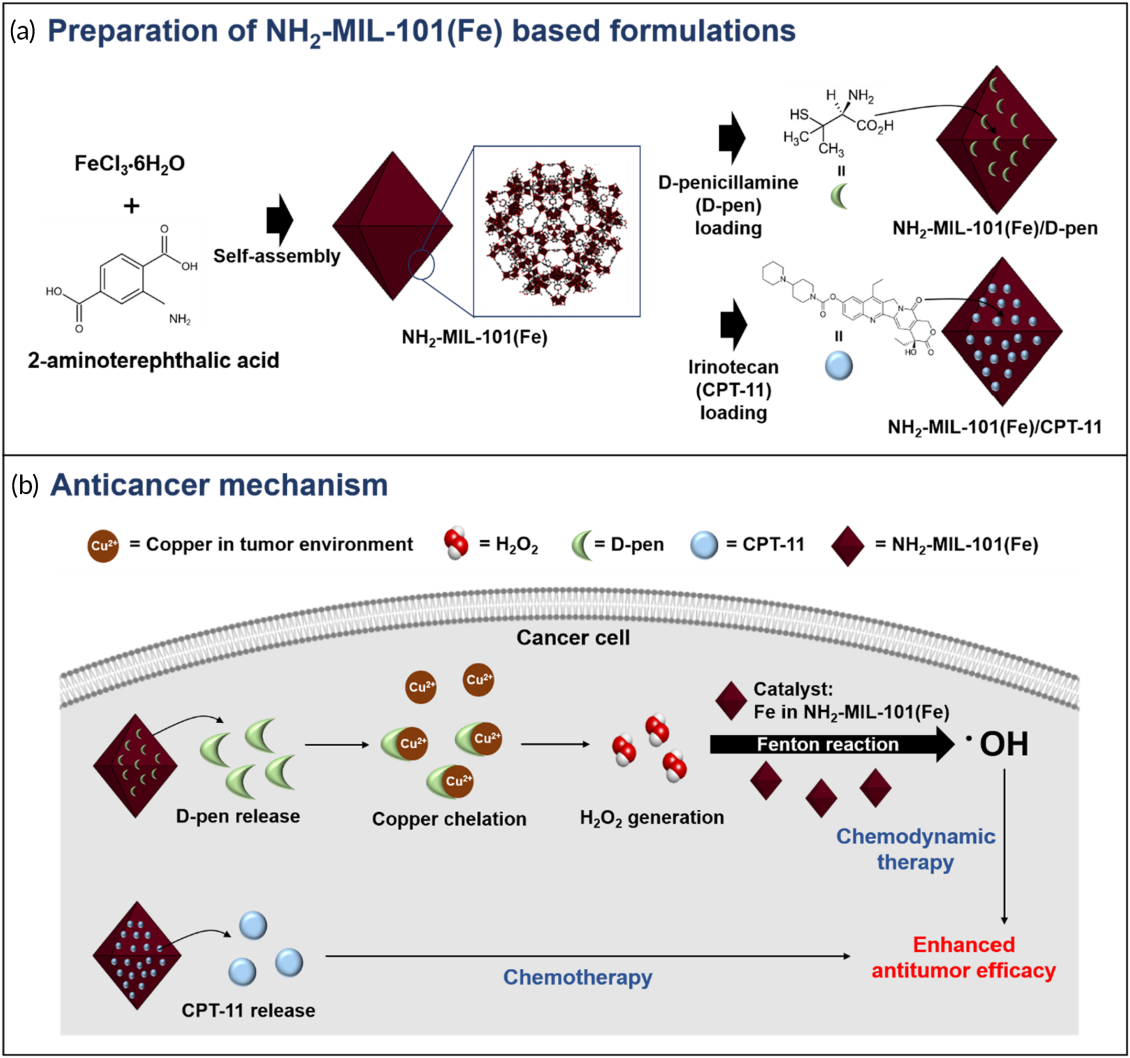
Schematic description of the NH_2_‐MIL‐101(Fe)‐based formulations. (a) Preparation of the NH_2_‐MIL‐101(Fe)/d‐pen and NH_2_‐MIL‐101(Fe)/CPT‐11. (b) Anticancer mechanism of the combined formulation of the NH_2_‐MIL‐101(Fe)/d‐pen and NH_2_‐MIL‐101(Fe)/CPT‐11

Cancer‐specific H_2_O_2_ generation followed by the Fenton reaction can be considered an advantageous strategy for safer and more efficient CDT. Therefore, we suggest a Fe‐containing metal–organic framework (MOF), that is, the NH_2_‐MIL‐101(Fe), to be loaded with d‐penicillamine (d‐pen) for CDT (Scheme 1). In this study, we focused on Cu(II) in cancer cells as a cancer‐specific endogenous component because its level is known to be significantly higher (>4 times) in cancer cells than that in normal cells.^23^, ^24^, ^25^ NH_2_‐MIL‐101(Fe) is composed of biocompatible materials, a metal cluster of Fe with an organic ligand of 2‐aminoterephtalic acid, and it possesses a highly porous structure with a large specific surface area (up to 2300 m^2^).^26^, ^27^ d‐pen, a Cu chelator, already approved for clinical use,^28^ could be loaded into NH_2_‐MIL‐101(Fe) pores to produce NH_2_‐MIL‐101(Fe)/d‐pen, from which d‐pen could be released in a sustained manner. The released d‐pen can then chelate Cu(II) most selectively among other divalent metal ions,^28^ which reduces Cu(II) to Cu(I), and oxidizes d‐pen to d‐pen disulfide.^29^, ^30^ This process produces H_2_O_2_ specifically to cancer cells as the reaction depends on Cu, which is highly expressed in cancer environments.^25^, ^31^ With the presence of Fe in NH_2_‐MIL‐101(Fe), the generated H_2_O_2_ would subsequently be disproportionated via the Fenton reaction, producing highly toxic ^•^OH.

In this study, we also examined the synergistic effects of CDT and chemotherapy. We separately prepared NH_2_‐MIL‐101(Fe) loaded with the anticancer drug irinotecan (CPT‐11) to produce NH_2_‐MIL‐101(Fe)/CPT‐11, which was employed together with NH_2_‐MIL‐101(Fe)/d‐pen (Scheme 1). This combined formulation would generate ^•^OH and release CPT‐11 in a sustained manner, which could improve the cytotoxic effect on cancer cells. For the suggested formulations, we evaluated the in vitro anticancer efficacy on the breast cancer cell line MCF‐7. The in vivo evaluations were performed in MCF‐7 xenograft animal models, where the formulations were administered via intratumoral injections.

## 2 MATERIALS AND METHODS

### 2.1 Materials

Fe(III) chloride hexahydrate (FeCl_3_·6H_2_O; 98%), 2‐aminoterephthalic acid (BDC‐NH_2_; 99%), *N*,*N*‐dimethylformamide (DMF; anhydrous, 99.8%), d‐penicillamine (d‐pen; 98%–101%), 4‐chloro‐7‐nitrobenzofurazan (NBD‐CL; 98%), copper(II) sulfate (CuSO_4_; >99%), formic acid (>98%) and 2′‐7′‐dichlorofluorescein diacetate (DCFH‐DA) were purchased from Sigma‐Aldrich (St. Louis, MO, USA). Methanol (>96%), ethanol (>96%), ammonium acetate (98%), sodium borate (Na_2_B_4_O_7_), and hydrogen peroxide (H_2_O_2_; 30%–35%) were obtained from Daejung Chemicals (Siheung, Korea). 3,3′,5,5′‐Tetramethylbenzidine (TMB; >98%) and acetonitrile (ACN, 99.9%) were purchased from TCI (Chuo‐ku, Japan) and J.T. Bakers (Pittsburgh, PA, USA), respectively. Irinotecan (CPT‐11; >99%) was obtained from LC Laboratories (Woburn, MA, USA). Phosphate‐buffered saline (PBS, pH 7.4) and Dulbecco's Modified Eagle's Medium (DMEM) were obtained from the Seoul National University Biomedical Research Institute (Seoul, Korea) and Welgene (Gyeongsan, Korea), respectively. Fetal bovine serum (FBS) and penicillin–streptomycin (10,000 U/ml) were purchased from Thermo Fisher Scientific (Waltham, MA, USA).

### 2.2 Preparation of NH_2_‐MIL‐101(Fe) and its formulations

NH_2_‐MIL‐101(Fe) was synthesized by the conventional solvothermal method following the reported procedures with slight modifications.^32^ Briefly, 1.35 g (4.994 mmol) of FeCl_3_·6H_2_O and 0.45 g (2.484 mmol) of BDC‐NH_2_ were dissolved in 30 ml of DMF, which was autoclaved for 24 h at 110°C. The resultant product was then purified by washing with DMF and methanol several times and dried at 70°C for 12 h. Subsequently, the solvent was further removed in a vacuum oven at 160°C for 12 h to produce the dehydrated NH_2_‐MIL‐101(Fe). In this work, we prepared the NH_2_‐MIL‐101(Fe) loaded with d‐pen, CPT‐11, or calcein to produce the NH_2_‐MIL‐101(Fe)/d‐pen, NH_2_‐MIL‐101(Fe)/CPT‐11, or NH_2_‐MIL‐101(Fe)/calcein, respectively. For this, 50 mg d‐pen, CPT‐11 or calcein was first dissolved in 10, 50, or 10 ml of DI water, respectively. To this solution, 50 mg of dehydrated NH_2_‐MIL‐101(Fe) was added with continuous stirring at 100 rpm for 48 h. After that, the suspension was filtered through a 200 nm nylon membrane filter (Hyundai Micro, Seoul, Korea). The resulting product was washed with ethanol three times and dried at 70°C.

### 2.3 Characterizations

X‐ray diffraction (XRD) data were acquired on an x‐ray diffractometer (SmartLab, Rigaku, Japan) at 3 kW using monochromatic Cu‐Kα radiation in the range of 5°–30°. Fourier transform infrared (FTIR) spectra were obtained using a TENSOR27 spectrophotometer (Bruker, Billerica, MA, USA) over the range of 4000–400 cm^−1^ with a resolution of 4 cm^−1^ at room temperature. The surface area, pore size, and pore volume were determined using a surface area and porosity analyzer (BELSOPR‐mini II, Microtrac‐Bel, Osaka, Japan). Prior to measurements, samples were degassed at 120°C for 12 h under vacuum conditions. Hydrodynamic size and zeta potential were measured using dynamic light scattering (DLS; ELS‐1000ZS, Otsuka Electronics, Osaka, Japan). The size and morphology were assessed using the scanning electron microscopy (SEM; JEOL‐7800F, JEOL, Tokyo, Japan) and transmission electron microscopy (TEM; JEM‐2100, JEOL, Tokyo, Japan).

To measure the amount of encapsulated d‐pen, CPT‐11, or calcein, 3 mg of NH_2_‐MIL‐101(Fe)/d‐pen, NH_2_‐MIL‐101(Fe)/CPT‐11, or NH_2_‐MIL‐101(Fe)/calcein was immersed in 50 ml of DI water, respectively, and stirred at 100 rpm at 37°C for 2 days to fully extract the encapsulated compound. The suspension was then centrifuged at 10,000 rpm and the supernatant was collected for analysis. To assess the loading amount of d‐pen, 4‐chloro‐7‐nitrobenzofurazan (NBD‐CL) was used as a probe to react with the amine groups of d‐pen.^33^ For this purpose, a buffered solution was prepared by mixing the aqueous solutions of 0.1 M NaOH and 0.025 M Na_2_B_4_O_7_ (v/v = 22.6:100). Then, 1 ml of the buffered solution was mixed with 1.5 ml of a 15 mM NBD‐CL solution prepared in MeOH, to which 1 ml of the collected supernatant containing d‐pen was added. After incubation for 30 min at room temperature, the reaction solution was measured spectrophotometrically at 486 nm (UV‐1800; Shimadzu, Kyoto, Japan) to determine the amount of d‐pen. To measure the loading amount of CPT‐11, the collected supernatant containing CPT‐11 was analyzed by high‐performance liquid chromatography (HPLC; Agilent 6120 systems, Agilent Technologies, Santa Clara, CA, USA) equipped with a Diamonsil C18 column (4.6 × 150 mm, 5 μm pore, Dikma, Lake Forest, CA, USA). A mobile phase composed of acetonitrile and 0.1% formic acid (v/v = 60:40) was pumped at a rate of 0.7 ml/min. A peak was detected at a wavelength of 360 nm.^34^ To measure the loading amount of calcein, the collected supernatant containing calcein was assayed spectrophotometrically at 495 nm using a UV–Vis spectrophotometer (UV‐1800; Shimadzu).^35^

### 2.4 In vitro release study

To examine the release profile of d‐pen, CPT‐11, or calcein, 5 mg of NH_2_‐MIL‐101(Fe)/d‐pen, NH_2_‐MIL‐101(Fe)/CPT‐11, or NH_2_‐MIL‐101(Fe)/calcein was dispersed in 1, 5, or 1 ml of PBS (pH 5.5) in a dialysis bag (3.5 kDa MWCO, SnakeSkin Dialysis Tubing, Thermo Fisher Scientific), respectively, which were then immersed in 4, 20, or 4 ml of PBS (pH 5.5), respectively. The sample was incubated at 37°C at 100‐rpm agitation in a shaking incubator (SI‐600R; Jeio Tech, Daejeon, Korea). At scheduled times, 1, 5, or 1 ml of the supernatant was extracted for NH_2_‐MIL‐101(Fe)/d‐pen, NH_2_‐MIL‐101(Fe)/CPT‐11, or NH_2_‐MIL‐101(Fe)/calcein, respectively, and an equal volume of fresh medium was added. The obtained media were assessed as described above to measure the concentrations of d‐pen, CPT‐11, or calcein.

### 2.5 In vitro study on ^•^ OH generation

To assess the capacity of ^•^OH generation, the formulations were each tested in a medium of pH 5.5 PBS containing 5 μM cupric sulfate, that is, a slightly acidic and Cu‐containing medium, mimicking the cancer environment.^36^ Thus, 0.5 mg of d‐pen, 4.5 mg of NH_2_‐MIL‐101(Fe), or 5 mg of NH_2_‐MIL‐101(Fe)/d‐pen, containing an equivalent amount of d‐pen and NH_2_‐MIL‐101(Fe), was added to 1 ml of the medium in a dialysis bag. The bag was immersed in 4 ml of the medium containing 1 mg/mL TMB, which was employed as a colorimetric probe for ^•^OH analysis.^37^, ^38^ The whole medium was then incubated at 37°C under 100‐rpm agitation in a shaking incubator. At each scheduled time, 1 ml of the supernatant was extracted and spectrophotometrically assessed at 652 nm (UV‐1800) to measure the amount of ^•^OH. To examine the Cu chelation property, 0.1 ml of the supernatant was also assessed using a Cu assay kit (KA1615; Abnova, Taipei, Taiwan) following the manufacturer's protocol. The experiments described above were repeated using 2.5 and 10 mg NH_2_‐MIL‐101(Fe)/d‐pen to examine the effect of concentration.

### 2.6 Cellular uptake assessment

To examine the intracellular uptake profile, we used NH_2_‐MIL‐101(Fe)/calcein, that is, NH_2_‐MIL‐101(Fe) loaded with calcein, a fluorescent probe.^39^, ^40^ MCF‐7 cells were seeded in a six‐well plate at a density of 5 × 10^5^ cells/well and cultured in the medium of DMEM for 24 h at 37°C with 5% CO_2_. The cells were then washed with a fresh medium and incubated with a suspension of NH_2_‐MIL‐101(Fe)/calcein at varying concentrations (50, 200, 500, and 1000 μg/ml) for 0.5, and 1 h at 37°C with 5% CO_2_, respectively. The cells were thoroughly washed three times with PBS containing 1% FBS to remove the residual NH_2_‐MIL‐101(Fe)/calcein. Then, 2 ml of trypsin was added to each well and incubated for 5 min to detach adherent cells. Subsequently, the cell suspension was centrifuged for 3 min at 1200 rpm, and the collected cells were suspended in 500 μl of PBS and analyzed with a flow cytometer (FACSymphony™ A5, BD Biosciences, Franklin Lakes, NJ, USA) using BD FACSDiva™ 8.0 software.

### 2.7 In vitro cytotoxicity evaluation

The in vitro cytotoxicity of NH_2_‐MIL‐101(Fe) was assessed in MCF‐7 and L929 cells by using a water‐soluble tetrazolium salt‐based cell viability assay (EZ‐Cytox; Daeil Lab Service, Korea).^41^ Briefly, MCF‐7 or L929 cells were seeded into a 96‐well cell plate at a density of 1.5 × 10^4^ cells/well in DMEM or RPMI containing 10% FBS and 1% penicillin–streptomycin solution, respectively, which was incubated at 37°C with 5% CO_2_ for 1 day. Next, the NH_2_‐MIL‐101(Fe) suspension prepared in the culture medium at various concentrations (10, 25, 50, 100, 250, 500, 1000, and 1500 μg/ml) was added to each well. After another 24 h of incubation, the culture medium was removed and replaced with an equal volume of fresh medium, to which 10 μl of EZ‐Cytox reagent was added and incubated for 2 h. The plate was then assessed by measuring the absorbance at wavelengths of 450 and 600 nm using a microplate reader (SpectraMax 190 Microplate Reader; Molecular Devices, San Jose, CA). Cell viability was calculated using the following equation: Cell viability (%) = (absorbance at 450 nm of the treated well – absorbance at 600 nm of the treated well)/(absorbance at 450 nm of the untreated control well – absorbance at 600 nm of the untreated control well) × 100.^42^

### 2.8 Assessment of intracellular ^•^ OH generation

MCF‐7 cells were seeded in a six‐well plate at a density of 3 × 10^5^ cells/well and cultured for 24 h at 37°C with 5% CO_2_. Subsequently, a d‐pen solution (666 μM), a suspension of NH_2_‐MIL‐101(Fe) (900 μg/ml), or a suspension of NH_2_‐MIL‐101(Fe)/d‐pen (1000 μg/ml) containing an equivalent amount of d‐pen and NH_2_‐MIL‐101(Fe) was added to each well and incubated for 6 h. Afterward, the cells were washed with fresh medium and incubated with 2 ml of 10 μM of DCFH‐DA for 30 min. DCFH‐DA was employed as a fluorescent probe for ^•^OH detection, as it produced fluorescent 2′‐7′‐dichlorofluorescein (DCF) when reacted with ^•^OH.^43^ Thus, the treated cells were examined under a confocal microscope (CLSM; TCS SP8 STED CW, Leica Microsystems, Wetzlar, Germany) at excitation and emission wavelengths of 488 and 530 nm, respectively. To examine the effect of concentration, the experiments described above were repeated with a suspension of 500 μg/ml NH_2_‐MIL‐101(Fe)/d‐pen, containing an equivalent amount of 333 μM d‐pen.

### 2.9 In vitro anticancer effect evaluation

We examined the anticancer efficacy of NH_2_‐MIL‐101(Fe)/d‐pen using MCF‐7 cells, which were seeded in a 96‐well plate at a density of 1.5 × 10^4^ cells/well and then incubated for 24 h. Then, the cells were treated with an aqueous solution of d‐pen at varying concentrations (5, 17, 33, 83, 167, 333, and 666 μM), a suspension of NH_2_‐MIL‐101(Fe) at varying concentrations (8, 23, 45, 113, 225, 450, and 900 μg/ml), and a suspension of NH_2_‐MIL‐101(Fe)/d‐pen at varying concentrations (9, 25, 50, 125, 250, 500, and 1000 μg/ml) that are equivalent to each of the corresponding concentrations of a d‐pen solution and NH_2_‐MIL‐101(Fe) suspension, respectively. After incubation for 48 h, the medium was replaced with an equal volume of fresh medium and 10 μl of EZ‐Cytox reagent was added and incubated for 2 h. Cell viability was calculated using a previously described equation.

To examine anticancer efficacy of the formulation combined with an anticancer drug, CPT‐11, we repeated the experiments described above, using the following formulations: an aqueous solution of CPT‐11, a suspension of NH_2_‐MIL‐101(Fe)/CPT‐11 only, a mixture of NH_2_‐MIL‐101(Fe)/d‐pen suspension and CPT‐11 solution (i.e., NH_2_‐MIL‐101(Fe)/d‐pen + CPT‐11), or a mixture of both NH_2_‐MIL‐101(Fe)/d‐pen and NH_2_‐MIL‐101(Fe)/CPT‐11 suspensions (i.e., NH_2_‐MIL‐101(Fe)/d‐pen + NH_2_‐MIL‐101(Fe)/CPT‐11). For each formulation, the equivalent CPT‐11 concentrations were 0.05, 1, 5, 10, and 20 μM. For NH_2_‐MIL‐101(Fe)/d‐pen + CPT‐11 and NH_2_‐MIL‐101(Fe)/d‐pen + NH_2_‐MIL‐101(Fe)/CPT‐11, NH_2_‐MIL‐101(Fe)/d‐pen concentration was fixed at 500 μg/ml.

### 2.10 In vivo anticancer efficacy

All animal experimental protocols were approved by the Institutional Animal Care and Use Committee (IACUC no. 21‐0063‐S1A0) of the Seoul National University Hospital Biomedical Research Institute. BALB/c nude mice were housed in a pathogen‐free facility with controlled environments: temperature, 21 ± 1°C; humidity, 55% ± 1%; light/dark cycle, 12 h/12 h.

In this study, we utilized 7‐week‐old tumor‐bearing BALB/c nude mice to examine the in vivo anticancer efficacy.^44^ For this, 100 μl of MCF‐7 cell suspension (1 × 10^7^ cells) prepared in Matrigel® (Corning, New York, USA) was injected subcutaneously into the flank. After 10–14 days, mice with an average tumor size equal to and over 100 mm^3^ were selected and randomly assigned to seven distinct treatment groups (*n* = 4 per group): (1) no treatment (i.e., saline injections) (2) NH_2_‐MIL‐101(Fe) (i.e., intratumoral injections of a NH_2_‐MIL‐101(Fe) suspension), (3) NH_2_‐MIL‐101(Fe)/d‐pen (i.e., intratumoral injections of a NH_2_‐MIL‐101(Fe)/d‐pen suspension), (4) CPT‐11 (i.e., intratumoral injections of a CPT‐11 solution), (5) NH_2_‐MIL‐101(Fe)/CPT‐11 (i.e., intratumoral injections of a NH_2_‐MIL‐101(Fe)/CPT‐11 suspension), (6) NH_2_‐MIL‐101(Fe)/d‐pen + CPT‐11 (i.e., intratumoral injections of a mixture of NH_2_‐MIL‐101(Fe)/D‐pen suspension and CPT‐11 solution), and (7) NH_2_‐MIL‐101(Fe)/d‐pen + NH_2_‐MIL‐101(Fe)/CPT‐11 (i.e., intratumoral injections of a mixture of both NH_2_‐MIL‐101(Fe)/d‐pen and NH_2_‐MIL‐101(Fe)/CPT‐11 suspensions). The formulation was injected once every 2 days for 18 days, and for each injection, the corresponding doses of d‐pen, CPT‐11, and NH_2_‐MIL‐101(Fe) were 3.75, 5, and 47.65 mg/kg, respectively.

Tumor volume and body weight were measured at scheduled times during treatment. The tumor volume was measured at the outside skin over the tumor using a Vernier caliper (CD‐15APX, Mitutuyo Corporation, Sakado, Japan) and calculated according to the formula: *V* = *d* ^2^ × *D*/2 (where *d* and *D* are the shortest and longest diameter of the tumor, respectively).^45^ At the endpoint of the treatment (18 days after the first treatment), the mice were sacrificed by CO_2_ asphyxiation, and tumors were dissected. The biopsied tumor tissue was then fixed in 4% neutral buffered formalin, embedded in paraffin, which was sectioned in 4 μm‐thick slices to be embedded on a tissue slide for histopathology and immunofluorescence analysis. The tissue slide was then stained with hematoxylin and eosin (H&E) and terminal deoxynucleotidyl transferase dUTP nick‐end labeling (TUNEL). For H&E staining, the slides were dipped in a hematoxylin solution for 10 min and washed with DI water and a mixed solution of 0.3% HCl and 70% ethanol. The slides were then immersed in eosin Y solution for 1 min and dehydrated with xylene and ethanol. For TUNEL staining, slides were stained using a kit (No. 11684817910; Roche, Basel, Switzerland) according to the manufacturer's protocol. Images of the stained slides were obtained at 40× magnification using an optical microscope (ECLIPSE Ts2, Nikon, Tokyo, Japan). For each analysis, four tissue slides were assessed from each animal, and thus, a total of 16 tissue slides were examined for each animal group. All tissue images were evaluated by a professional pathologist (C. L.) in a blinded manner.

### 2.11 Statistical analysis

Data are reported as mean ± standard deviation. The values of the cell viability and tumor volume were statistically analyzed using the Mann–Whitney U test. (GraphPad Prism 7.0, GraphPad Software, San Diego, USA), where *p* < 0.05 was considered statistically different (*p < 0.05, ** *p* < 0.01).

## 3 RESULTS

### 3.1 Characterization of NH_2_‐MIL‐101(Fe) and formulations

**FIGURE 1:**
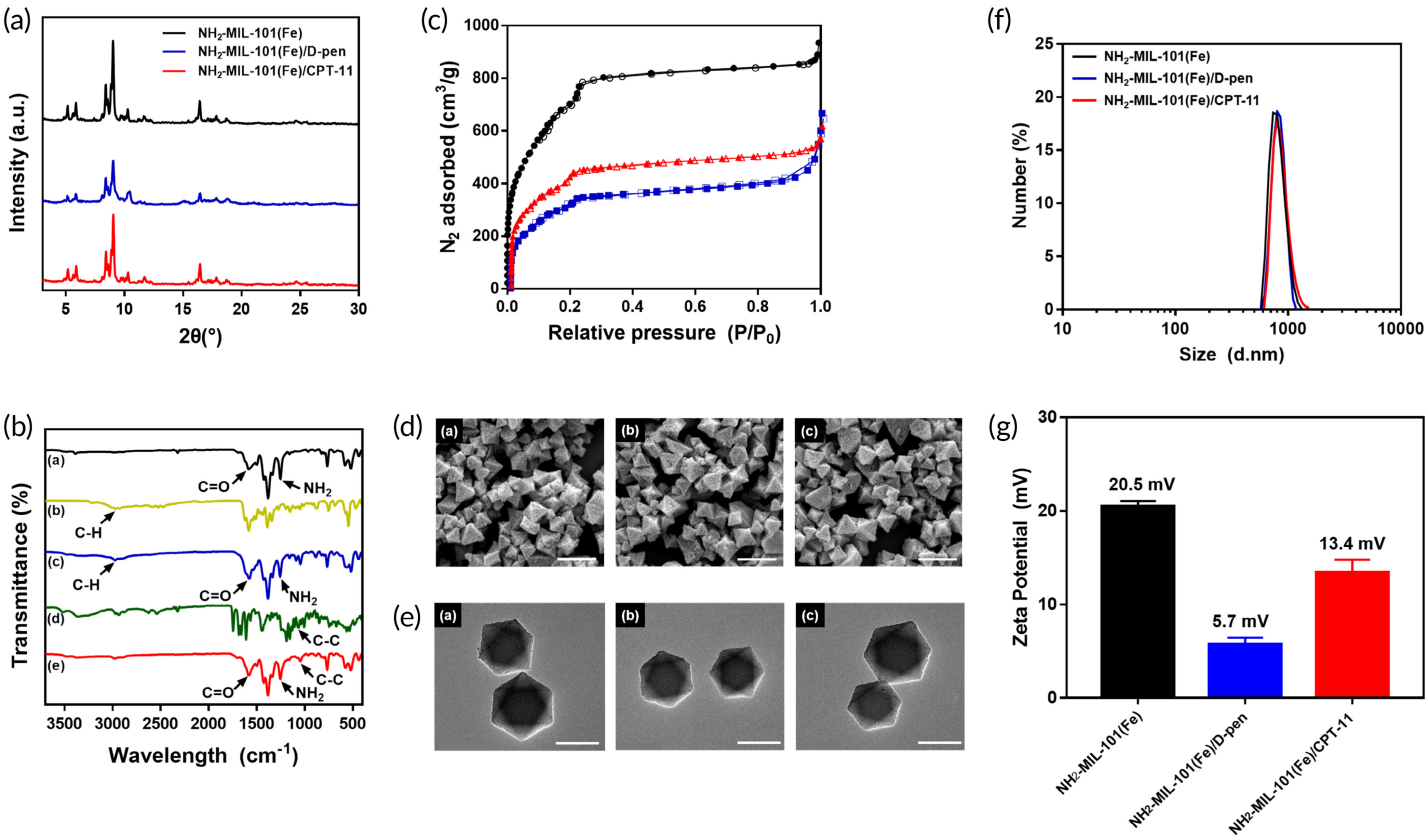
(a) Powder x‐ray diffraction (PXRD) patterns of NH_2_‐MIL‐101(Fe), NH_2_‐MIL‐101(Fe)/d‐pen and NH_2_‐MIL‐101(Fe)/CPT‐11. (b) FTIR spectra of (a) NH_2_‐MIL‐101(Fe), (b) d‐pen, (c) NH_2_‐MIL‐101(Fe)/d‐pen, (d) CPT‐11 and (e) NH_2_‐MIL‐101(Fe)/CPT‐11. (c) N_2_ isotherm profiles of NH_2_‐MIL‐101(Fe), NH_2_‐MIL‐101(Fe)/d‐pen and NH_2_‐MIL‐101(Fe)/CPT‐11. (●) NH_2_‐MIL‐101(Fe) adsorption, (○) NH_2_‐MIL‐101(Fe) desorption, (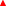) NH_2_‐MIL‐101(Fe)/CPT‐11 adsorption, (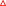) NH_2_‐MIL‐101(Fe)/CPT‐11 desorption, (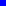) NH_2_‐MIL‐101(Fe)/d‐pen adsorption and (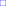) NH_2_‐MIL‐101(Fe)/d‐pen desorption. (d) Scanning electron microscopy (SEM) and (e) transmission electron microscopy (TEM) images of (a) NH_2_‐MIL‐101(Fe), (b) NH_2_‐MIL‐101(Fe)/d‐pen and (c) NH_2_‐MIL‐101(Fe)/CPT‐11. The scale bars are (d) 1 μm and (e) 500 nm. (f) Size distributions and (g) zeta potentials of NH_2_‐MIL‐101(Fe), NH_2_‐MIL‐101(Fe)/d‐pen, and NH_2_‐MIL‐101(Fe)/CPT‐11. Error bars represent the standard deviation (*n* = 3).

NH_2_‐MIL‐101(Fe) was synthesized using the solvothermal method, in which d‐pen or CPT‐11 were loaded into the pores of NH_2_‐MIL‐101(Fe) via physical adsorption to prepare NH_2_‐MIL‐101(Fe)/d‐pen or NH_2_‐MIL‐101(Fe)/CPT‐11, respectively. As shown in the powder x‐ray diffraction (PXRD) patterns in Figure 1a, NH_2_‐MIL‐101(Fe) exhibits well‐defined diffraction patterns owing to its crystalline structure, as previously reported.^46^ The PXRD patterns did not change for NH_2_‐MIL‐101(Fe)/d‐pen and NH_2_‐MIL‐101(Fe)/CPT‐11, suggesting that the crystallinity of NH_2_‐MIL‐101(Fe) was retained after compound loading. As shown in Figure 1b, the FTIR spectrum of NH_2_‐MIL‐101(Fe) exhibited characteristic peaks at 1581 and 763 cm^−1^ due to C=O bonding in the carboxylate and C—H bending vibrations of benzene, respectively.^47^, ^48^ For d‐pen, the peak at 2970 cm^−1^ was ascribed to C—H stretching of the methyl group in d‐pen.^49^ The FTIR spectra of CPT‐11 showed bands at 1037 cm^−1^ from the C—C stretching vibrations of bipiperidine.^50^ For NH_2_‐MIL‐101(Fe)/d‐pen and NH_2_‐MIL‐101(Fe)/CPT‐11, the characteristic peaks from both NH_2_‐MIL‐101(Fe) and each of the loaded compounds overlapped without any apparent shift, implying that their chemical structures were not altered after encapsulation. Figure 1c shows the N_2_ adsorption–desorption isotherms of NH_2_‐MIL‐101(Fe), NH_2_‐MIL‐101(Fe)/d‐pen, and NH_2_‐MIL‐101(Fe)/CPT‐11. The N_2_ isotherms achieved can be considered type 1 according to the International Union of Pure and Applied Chemistry (IUPAC) classification^51^ confirming that the microporous structures were retained before and after compound loading. As shown in Table 1, the surface area and total pore volume of NH_2_‐MIL‐101(Fe) decreased after d‐pen or CPT‐11 loading because of the molecules being encapsulated in the pores of NH_2_‐MIL‐101(Fe). These results were consistent with those reported previously.^46^ The loading amounts of d‐pen and CPT‐11 were 101 ± 3 and 254 ± 5 μg/mg, respectively.

**TABLE 1:** Characterization results of the NH_2_‐MIL‐101(Fe), NH_2_‐MIL‐101(Fe)/d‐pen, and NH_2_‐MIL‐101(Fe)/CPT‐11

	NH_2_‐MIL‐101(Fe)	NH_2_‐MIL‐101(Fe)/d‐pen	NH_2_‐MIL‐101(Fe)/CPT‐11
Surface area (m^2^/g)^a^	2252.1	1305.6	1561.2
Pore volume (cm^3^/g)	1.38	0.97	0.93
Loading amount (μg/mg)	‐	101 ± 3 (d‐pen)	254 ± 5 (CPT‐11)

^a^ Surface area was calculated based on the Brunauer–Emmett–Teller (BET) method.^46^

The SEM and TEM images in Figure 1d,f show that most of synthesized NH_2_‐MIL‐101(Fe) (>90%) is a nano‐sized particle with an octahedral shape, as previously reported,^46^, ^52^ which did not change with NH_2_‐MIL‐101(Fe)/d‐pen and NH_2_‐MIL‐101(Fe)/CPT‐11 after compound loading. According to the DLS analysis (Figure 1f,g), the average particle sizes of NH_2_‐MIL‐101(Fe), NH_2_‐MIL‐101(Fe)/d‐pen, and NH_2_‐MIL‐101(Fe)/CPT‐11 were 723, 756, and 766 nm, respectively. The surface charge of the NH_2_‐MIL‐101(Fe) was 20.5 mV, which, however, decreased to 5.7 and 13.4 mV with the NH_2_‐MIL‐101(Fe)/d‐pen and NH_2_‐MIL‐101(Fe)/CPT‐11 as each of the loaded compounds was negatively charged.^53^, ^54^

### 3.2 In vitro compound release and ^•^ OH generation

**FIGURE 2:**
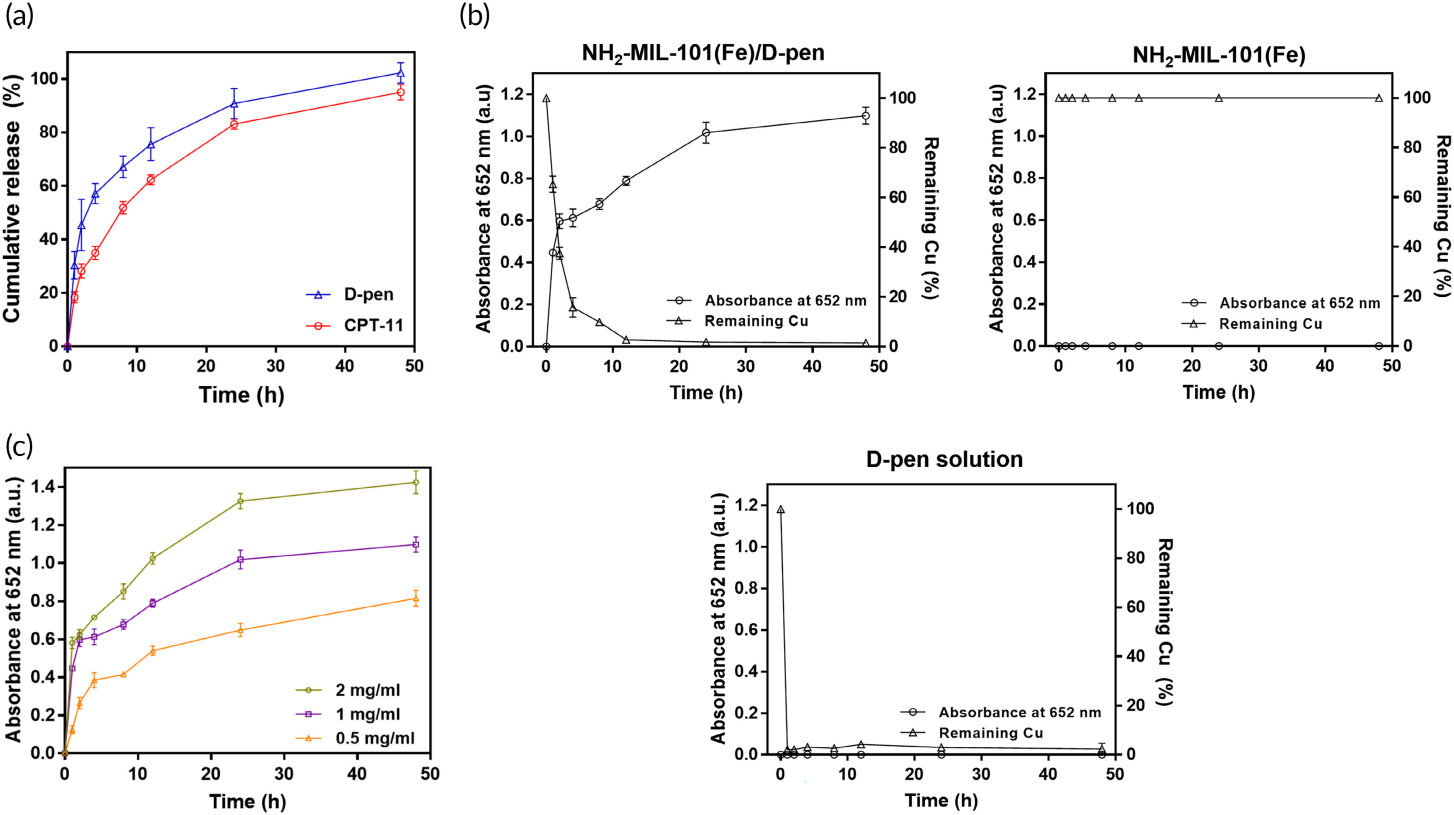
(a) In vitro release profiles of d‐pen and CPT‐11 from the NH_2_‐MIL‐101(Fe)/d‐pen and NH_2_‐MIL‐101(Fe)/CPT‐11, respectively. Error bars represent the standard deviation (*n* = 3). (b) In vitro profiles of ^•^OH generation and Cu chelation in the Cu‐containing medium of pH 5.5 PBS for NH_2_‐MIL‐101(Fe)/d‐pen, NH_2_‐MIL‐101(Fe) and d‐pen solution. (c) Profiles of ^•^OH generation from the NH_2_‐MIL‐101(Fe)/d‐pen at varying concentrations. The medium also contained a colorimetric probe, TMB, where the content of ^•^OH was measured spectrophotometrically at 652 nm. Error bars represent the standard deviation (*n* = 5).

The in vitro release profiles of d‐pen and CPT‐11 were obtained using NH_2_‐MIL‐101(Fe)/d‐pen and NH_2_‐MIL‐101(Fe)/CPT‐11, respectively, using the medium of pH 5.5 PBS to mimic the acidic environment of cancer.^55^ As shown in Figure 2a, both d‐pen and CPT‐11 were released in a sustained manner for up to 2 days, mainly by out‐diffusion of the compound encapsulated within the pores of NH_2_‐MIL‐101(Fe).^26^, ^56^ There was an initial burst release of d‐pen during the first 4 h possibly due to the presence of d‐pen distributed on the surface of NH_2_‐MIL‐101(Fe), which was not apparent with CPT‐11 due to its lower water solubility than that of d‐pen.^57^, ^58^

In this study, we hypothesized that H_2_O_2_ could be generated from Cu chelation by d‐pen released from NH_2_‐MIL‐101(Fe)/d‐pen, which would react with a Fe catalyst in NH_2_‐MIL‐101(Fe) to produce ^•^OH. To test this, NH_2_‐MIL‐101(Fe)/d‐pen was placed in pH 5.5 PBS containing 5 μM cupric sulfate as a simulated cancer environment.^36^ Under these conditions, the 3,3′,5,5′‐tetramethylbenzidine (TMB) chromogenic reaction was applied to detect light absorbance at 652 nm as an indicator of ^•^OH radical production.^37^ As shown in Figure 2b, when the NH_2_‐MIL‐101(Fe)/d‐pen was tested, the absorbance gradually increased during the first 48 h, indicating a cumulative increase in ^•^OH radicals in the medium. A gradual decrease in the amount of Cu was concurrently observed as d‐pen was slowly released to chelate Cu and produce H_2_O_2_, which in turn generated ^•^OH radicals via the Fenton reaction with Fe in NH_2_‐MIL‐101(Fe). After 12 h, almost all Cu was depleted, but the absorbance gradually increased because Cu in the d‐pen/Cu complex was still able to oxidize the d‐pen released continuously from NH_2_‐MIL‐101(Fe)/d‐pen (Figure 2a).^59^ When NH_2_‐MIL‐101(Fe) without d‐pen was tested (Figure 2b), almost no absorbance was detected and the amount of Cu in the medium was maintained without consumption. In a d‐pen solution without NH_2_‐MIL‐101(Fe), all Cu was depleted almost instantaneously because of free d‐pen molecules at the same dose in the medium; however, without the presence of an Fe catalyst, the absorbance was almost zero, that is, no ^•^OH radical production (Figure 2b). As shown in Figure 2c, the dose dependency of NH_2_‐MIL‐101(Fe)/d‐pen was apparent. At a fixed concentration of Cu in the medium, the amount of ^•^OH increased with increasing concentration of NH_2_‐MIL‐101(Fe)/d‐pen. When tested in pH 5.5 PBS without Cu ions, NH_2_‐MIL‐101(Fe)/d‐pen showed no absorbance (Figure S1), indicating that Cu chelation plays an essential role in H_2_O_2_ generation.

### 3.3 In vitro cell tests

**FIGURE 3:**
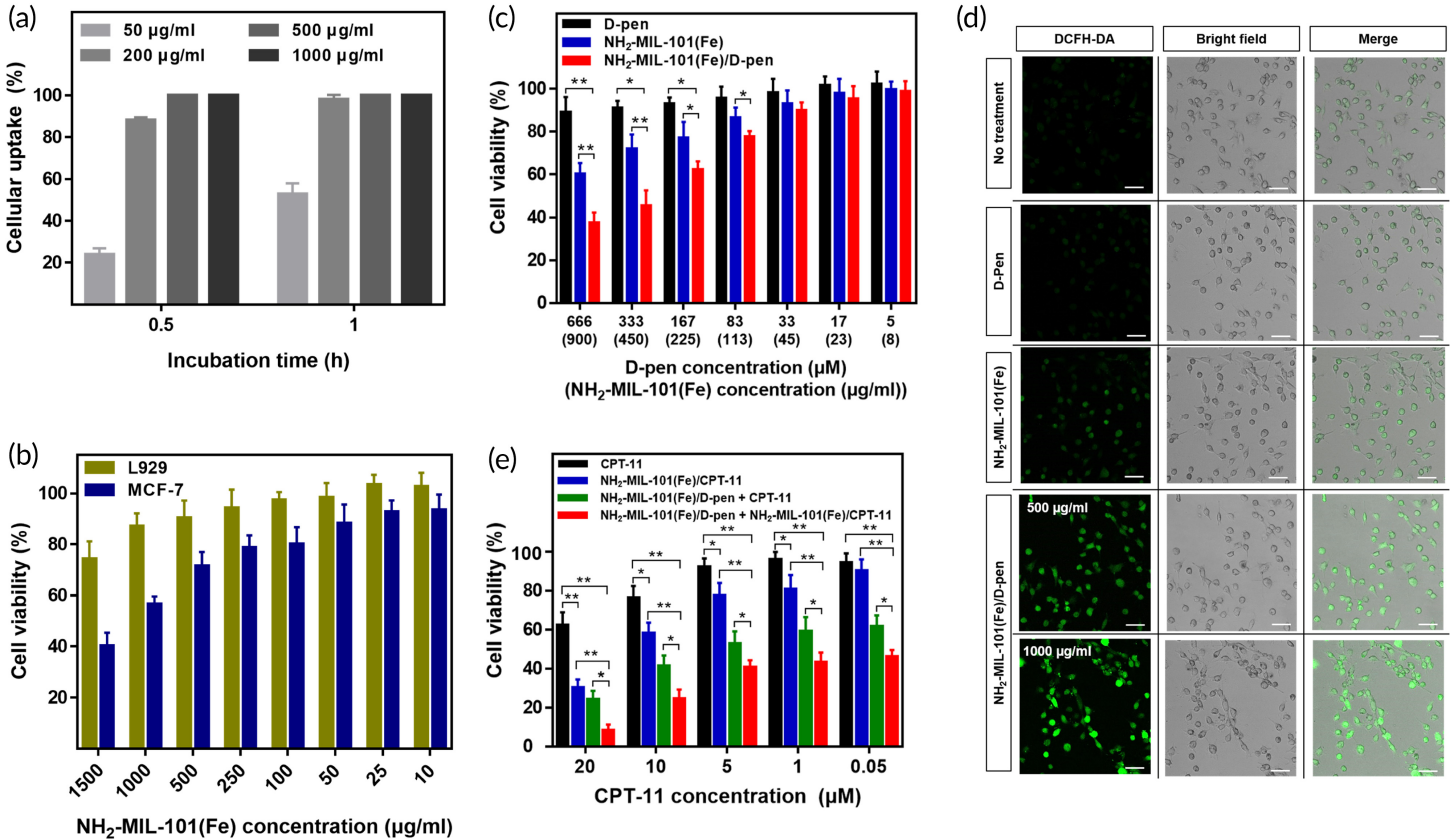
In vitro cell experimental results. (a) Intracellular uptake profiles of the NH_2_‐MIL‐101(Fe)/calcein using MCF‐7 cells measured by flow cytometry. Error bars represent the standard deviation (*n* = 3). (b) Cytotoxicity test results with the NH_2_‐MIL‐101(Fe) using L929 and MCF‐7 cells. Error bars represent the standard deviation (*n* = 6). (c) Antitumor effects on MCF‐7 cells. The tested formulations were d‐pen, NH_2_‐MIL‐101(Fe), and NH_2_‐MIL‐101(Fe)/d‐pen. Error bars represent the standard deviation (*n* = 6). *p < 0.05 and **p < 0.01. (d) Confocal fluorescence microscopic images of MCF‐7 cells treated with DCFH‐DA after exposure to a d‐pen solution, or suspension of NH_2_‐MIL‐101(Fe) or NH_2_‐MIL‐101(Fe)/d‐pen. Green fluorescence indicates the presence of ^•^OH. The scale bars are 10 μm. (e) Antitumor effects on MCF‐7 cells. The tested formulations were CPT‐11, NH_2_‐MIL‐101(Fe)/CPT‐11, NH_2_‐MIL‐101(Fe)/d‐pen + CPT‐11, and NH_2_‐MIL‐101(Fe)/d‐pen + NH_2_‐MIL‐101(Fe)/CPT‐11. Error bars represent the standard deviation (*n* = 6). *p < 0.05 and **p < 0.01

NH_2_‐MIL‐101(Fe) was used as a carrier to be engulfed into cancer cells and release the encapsulated compound. Thus, to determine the intracellular uptake profile, NH_2_‐MIL‐101(Fe)/calcein (loading amount = 308 ± 6 μg/mg) was applied to MCF‐7 cells using calcein as a fluorescent probe (Figures S2 and S3).^39^ As shown in Figure 3a, flow cytometry analysis revealed that NH_2_‐MIL‐101(Fe) could be delivered efficiently into cells within 1 h at concentrations equal to or above 200 μg/ml. Figure 3b shows cell viability after treatment with NH_2_‐MIL‐101(Fe). For the L929 cells, as representative of normal cells, NH_2_‐MIL‐101(Fe) exhibited negligible cytotoxicity at all tested concentrations. In contrast, when tested with MCF‐7 cancer cells, there was an observable decrease in cell viability with increasing NH_2_‐MIL‐101(Fe) concentration. It has been reported that the level of H_2_O_2_ is higher in cancer cells than that in normal cells,^60^ where even without d‐pen, the Fe in NH_2_‐MIL‐101(Fe) could still react as a catalyst and produce ^•^OH radicals to cause cytotoxicity.

The cytotoxic effect of NH_2_‐MIL‐101(Fe)/d‐pen on cancer cells was more prominent. As shown in Figure 3c, NH_2_‐MIL‐101(Fe)/d‐pen showed significantly lower cell viability at concentrations of 113 μg/ml or higher compared with NH_2_‐MIL‐101(Fe) at the same dose, which suggested the generation of extra H_2_O_2_ during Cu chelation with released d‐pen, thereby increasing the number of ^•^OH radicals by the Fenton reaction. This level of cytotoxicity was not observed in L929 cells, as Cu was not highly expressed in normal cells (Figure S4).^61^ The d‐pen is biocompatible,^30^ and it did not produce ^•^OH by itself (Figure 2b), thereby resulting in no apparent cytotoxicity. The confocal fluorescence microscopic images in Figure 3d further support the capacity for ^•^OH production with NH_2_‐MIL‐101(Fe)/d‐pen. The cancer cells were treated with DCFH‐DA; thus, a green fluorescence signal indicated the presence of ^•^OH.^43^ Weak fluorescence was observed in untreated cells because of the naturally present ^•^OH in cancer cells,^62^, ^63^ which did not change significantly for cells treated with d‐pen only, indicating no additional ^•^OH production. An increase in fluorescence intensity was observed when the cells were treated with NH_2_‐MIL‐101(Fe) only. However, it should be noted that cells treated with NH_2_‐MIL‐101(Fe)/d‐pen exhibited significantly stronger fluorescence, which became brighter as the NH_2_‐MIL‐101(Fe)/d‐pen concentration increased.

We then sought to determine the anticancer effects of a combination of CDT and chemotherapy. MCF‐7 cells were treated with four different formulations at varying doses of an anticancer drug, CPT‐11. As shown in Figure 3e, for all formulations, cell viability gradually decreased as the CPT‐11 concentration increased. However, compared with a bolus CPT‐11 solution, the NH_2_‐MIL‐101(Fe)/CPT‐11 exhibited a significantly higher cytotoxicity due to more efficient intracellular delivery and sustained drug release with the NH_2_‐MIL‐101(Fe)/CPT‐11 (Figures 2a and 3a). A synergistic effect was observed when NH_2_‐MIL‐101(Fe)/d‐pen was used. At the same concentration of CPT‐11, the CPT‐11 solution with NH_2_‐MIL‐101(Fe)/d‐pen was more cytotoxic than that with NH_2_‐MIL‐101(Fe)/CPT‐11 only. Notably, the anticancer effect was most prominent when NH_2_‐MIL‐101(Fe)/d‐pen and NH_2_‐MIL‐101(Fe)/CPT‐11 were combined. Both NH_2_‐MIL‐101(Fe)‐based particles appeared to be efficiently engulfed into cells, where ^•^OH radicals were produced and CPT‐11 was released in a sustained manner, resulting in a much‐improved anticancer effect.

### 3.4 In vivo anticancer efficacy

**FIGURE 4:**
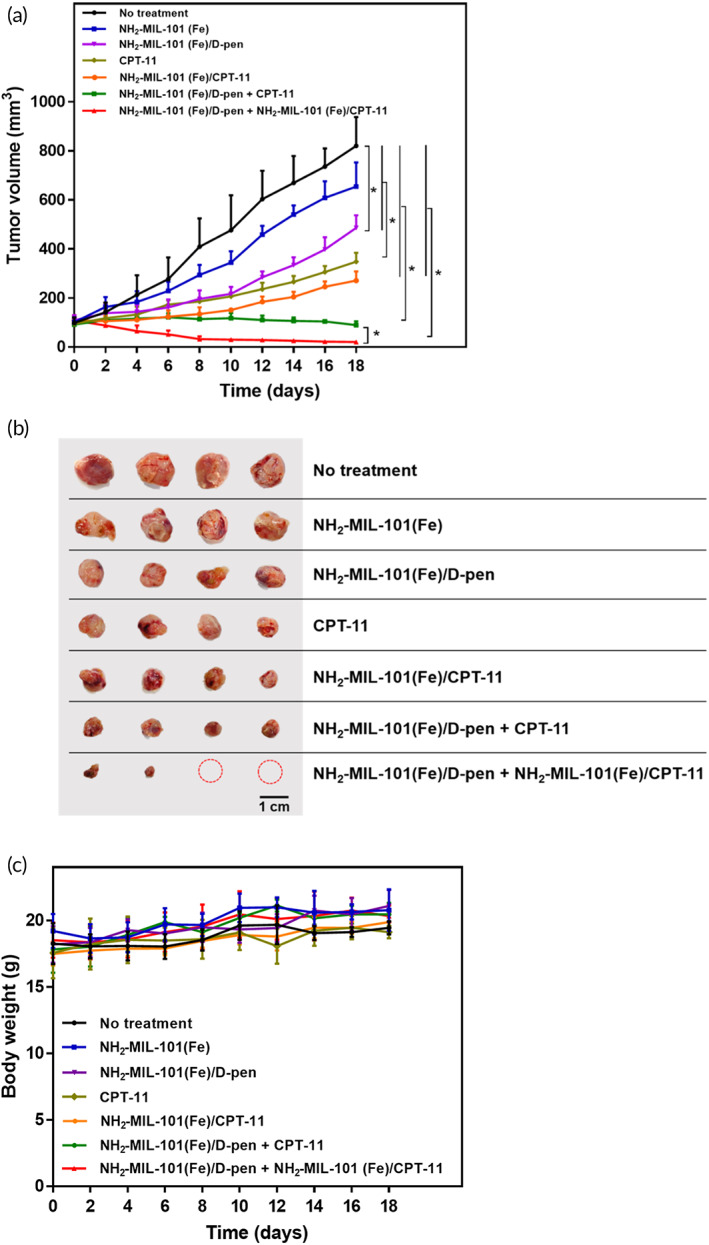
In vivo antitumor effects from seven different animal groups: (1) No treatment, (2) NH_2_‐MIL‐101(Fe), (3) NH_2_‐MIL‐101(Fe)/d‐pen, (4) CPT‐11, (5) NH_2_‐MIL‐101(Fe)/CPT‐11, (6) NH_2_‐MIL‐101(Fe)/d‐pen + CPT‐11, and (7) NH_2_‐MIL‐101(Fe)/d‐pen + NH_2_‐MIL‐101(Fe)/CPT‐11. (a) Tumor volume profiles. Error bars represent the standard deviation (*n* = 4). *p < 0.05 and **p < 0.01. b) Optical images of the whole tumor tissues biopsied at the end point of experiments (18 days). The scale bar is 1 cm. (c) Profiles of body weight during whole testing periods of 18 days. Error bars represent the standard deviation (*n* = 4).

The in vivo therapeutic efficacy of the formulations was evaluated in seven distinct groups of mice bearing MCF‐7 cells: (1) no treatment; and intratumoral injections of (2) NH_2_‐MIL‐101(Fe), (3) NH_2_‐MIL‐101(Fe)/d‐pen, (4) CPT‐11, (5) NH_2_‐MIL‐101(Fe)/CPT‐11, (6) NH_2_‐MIL‐101(Fe)/d‐pen + CPT‐11, and (7) NH_2_‐MIL‐101(Fe)/d‐pen + NH_2_‐MIL‐101(Fe)/CPT‐11. As shown in Figure 4a, the tumor volume dramatically increased in the No treatment group, as expected. The NH_2_‐MIL‐101(Fe) group only showed slight slowdown of tumor growth because even without the presence of d‐pen, endogenous H_2_O_2_ in cancer cells could still produce ^•^OH by the Fenton reaction with NH_2_‐MIL‐101(Fe) (Figure 3c). NH_2_‐MIL‐101(Fe)/d‐pen group showed a more suppressed tumor growth due to the increase in H_2_O_2_ by Cu chelation. The tumor growth was further suppressed in CPT‐11 group. However, in vivo, the difference in tumor growth was not significant between CPT‐11 and NH_2_‐MIL‐101(Fe)/CPT‐11 groups.

**FIGURE 5:**
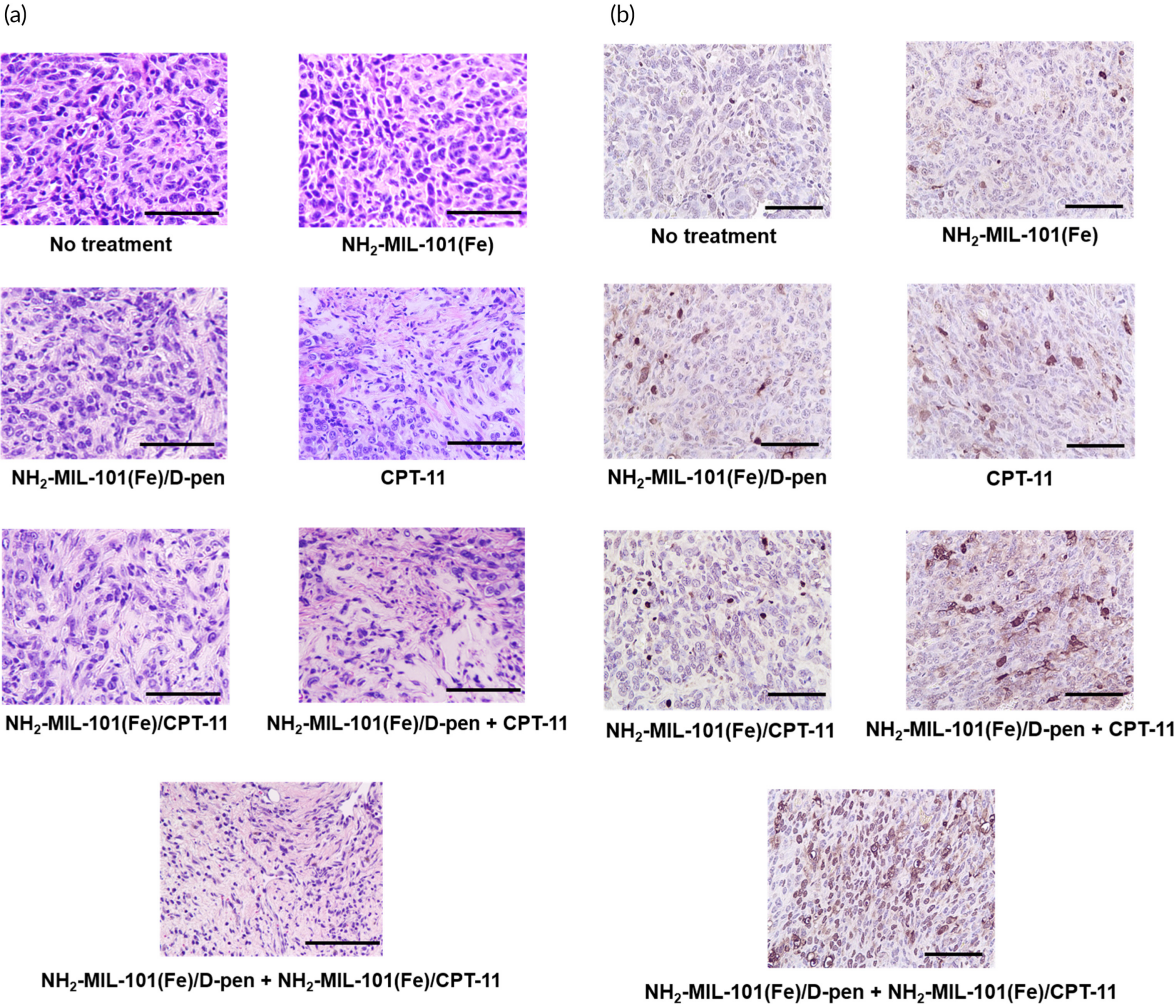
Representative images of (a) H&E‐ and (b) terminal deoxynucleotidyl transferase dUTP nick‐end labeling (TUNEL)‐stained tumor tissues biopsied at the end point of experiments (18 days). The scale bars are 50 μm.

An anticancer effect was evident even with a bolus CPT solution when injected together with NH_2_‐MIL‐101(Fe)/d‐pen. The average tumor volume did not increase significantly during the entire testing period, indicating a synergistic effect of CDT and chemotherapy. Notably, this was most prominent in NH_2_‐MIL‐101(Fe)/d‐pen + NH_2_‐MIL‐101(Fe)/CPT‐11 group, where the tumor volume even decreased under in vivo experimental conditions herein. In addition to the chemodynamic effect, more efficient intracellular delivery and sustained release of CPT‐11 boosted the anticancer ability with the combined formulation of NH_2_‐MIL‐101(Fe)/d‐pen and NH_2_‐MIL‐101(Fe)/CPT‐11. As shown in Figure 4b, the tumor tissues biopsied at the end of the experiments also revealed a remarkable decrease in volume in the NH_2_‐MIL‐101(Fe)/d‐pen + NH_2_‐MIL‐101(Fe)/CPT‐11 group compared with that of the other groups. During the entire testing period, there was almost no change in body weight for any of the animal groups, suggesting that the NH_2_‐MIL‐101(Fe)‐based formulations had no significant systemic side effects (Figure 4c). Histological analysis of the H&E‐stained tumor tissues further confirmed the anticancer effect of the combination of NH_2_‐MIL‐101(Fe)/d‐pen and NH_2_‐MIL‐101(Fe)/CPT‐11, showing the most severe damage, such as cell shrinkage and nuclear fragmentation (Figure 5a). TUNEL‐stained tumor tissues also exhibited maximum cell death in the NH_2_‐MIL‐101(Fe)/d‐pen + NH_2_‐MIL‐101(Fe)/CPT‐11 group (Figure 5b).

## 4 DISCUSSION

CDT has drawn a great deal of interest as a novel therapeutic modality for the treatment of cancer, where metal ions, such as Fe and Mn, are utilized as key catalysts in the Fenton reaction to decompose H_2_O_2_ and produce toxic radicals of ^•^OH to kill cancer cells.^64^ Conventional catalysts are often prepared in nanoparticles containing catalytic metals, which, however, are limited by their low aqueous stability and heterogeneous reactivity, resulting in inefficient CDT effects.^11^, ^13^ Moreover, the amount of endogenous H_2_O_2_ in tumors was not sufficient to produce a therapeutically effective amount of ^•^OH.^65^ To compensate for this, H_2_O_2_ has been additionally delivered or produced; however, the approach is not cancer specific, thereby uncontrolled exposure of toxic ^•^OH to the surrounding normal tissues.^21^

To address these issues, we propose an NH_2_‐MIL‐101(Fe)‐based formulation to enhance the anticancer effect of CDT (Scheme 1). NH_2_‐MIL‐101(Fe) is a MOF with high porosity, where a compound of interest can be encapsulated. More importantly, there are multiple Fe metal clusters in NH_2_‐MIL‐101(Fe) that serve as stable catalytic sites for the Fenton reaction. In this study, we focused on the high level of Cu in cancer cells as a means of generating more H_2_O_2_, an essential reagent of the Fenton reaction.^24^ Therefore, we employed a biocompatible Cu chelator, d‐pen,^30^ for loading into the pores of NH_2_‐MIL‐101(Fe).^26^ NH_2_‐MIL‐101(Fe)/d‐pen was an octahedral nanoparticle with multiple sharp edges (Figure 1d,e), which allowed for efficient intracellular uptake (Figure 3a).^66^, ^67^ Thus, inside the cancer cells, NH_2_‐MIL‐101(Fe)/d‐pen released d‐pen to chelate Cu and provide additional H_2_O_2_ (Figure 2b). This exogenous H_2_O_2_, as well as the endogenous one, went the Fenton reaction with Fe in NH_2_‐MIL‐101(Fe), producing cytotoxic ^•^OH (Figure 3c,d). Because of this strategy, the Fenton reaction with NH_2_‐MIL‐101(Fe) is cancer‐specific. In normal cells, there was no apparent cytotoxicity of the formulation owing to the very low levels of H_2_O_2_ and Cu (Figure 3b and Figure S4).^23^, ^68^

The anticancer efficacy was further improved when the formulation for CDT (i.e., NH_2_‐MIL‐101(Fe)/d‐pen) was combined with that of a chemotherapy drug, CPT‐11 (Figure 3c). In this work, each compound of interest was loaded into the NH_2_‐MIL‐101(Fe) separately to produce the NH_2_‐MIL‐101(Fe)/d‐pen and NH_2_‐MIL‐101(Fe)/CPT‐11, respectively, which were mixed together for a more reproducible loading and release profile. Owing to the properties of sustained drug release and efficient intracellular uptake, NH_2_‐MIL‐101(Fe)/d‐pen combined with NH_2_‐MIL‐101(Fe)/CPT‐11 exhibited higher cytotoxicity than that combined with bolus CPT‐11. Our in vivo results during a relatively short period of 18 days indeed exhibited the most prominent anticancer effect with the combined formulation of NH_2_‐MIL‐101(Fe)/d‐pen and NH_2_‐MIL‐101(Fe)/CPT‐11 (Figures 4 and 5), where the tumor decreased relative to its initial size and almost disappeared in a few animals. Notably, with NH_2_‐MIL‐101(Fe)/d‐pen only, the tumor growth was still suppressed, indicating an apparent efficacy of CDT aided by additional H_2_O_2_ generation via Cu chelation. Our formulation was composed of biocompatible materials^26^, ^30^; thus, it did not exhibit any sign of systemic toxicity (Figure 4c), which could be further supported by cancer‐specific ^•^OH generation with our current strategy (Figure 3b and Figure S4). After the completion of the reaction and drug release, almost all NH_2_‐MIL‐101(Fe) was expected to degrade into soluble small molecules of biocompatible ligands and metal ions (Figure S5).^69^ For this reason, the regimen of the current strategy could be repeated for a prolonged period in case a further treatment was needed. With the designated sizes of the NH_2_‐MIL‐101(Fe) herein,^70^ the formulation could be injected intravenously with a possible targeted delivery of therapeutic compounds.^71^, ^72^

In previous studies, ZIF‐8 and MIL‐100 MOFs were employed for CDT purposes, where the generation of exogenous H_2_O_2_ was based on glucose oxidase (GOx). Thus, ZIF‐8 was utilized as a carrier for the delivery of GOx and to produce ^•^OH, which needed to be loaded together with catalytic compounds for the Fenton reaction.^73^, ^74^, ^75^, ^76^ However, ZIF‐8 was degraded rapidly in hours in acidic cancer environments,^77^ and thus, the bioavailability of the encapsulated compounds was low, leading to a limited production of ^•^OH.^78^ MIL‐100 also worked as a carrier for the delivery of GOx, which, on the other hand, could also serve as a catalyst for the Fenton reaction by itself owing to the constituent metal clusters of Fe.^79^ However, the use of GOx is limited by its immunogenicity, low stability, and high cost.^80^ Furthermore, the substrate of GOx, for example, glucose, is ubiquitous in the body; thus, the Fenton reaction is not cancer specific, making the formulation susceptible to systemic side effects.

## 5 CONCLUSION

We suggest that NH_2_‐MIL‐101(Fe) be loaded with the biocompatible Cu chelator, d‐pen, for cancer‐specific CDT. NH_2_‐MIL‐101(Fe), a type of MOF, possesses a highly porous structure that can serve as a d‐pen carrier. NH_2_‐MIL‐101(Fe)/d‐pen can be fabricated using nanoparticles with sharp edges, which facilitate their intracellular uptake. Therefore, d‐pen can be released in a sustained manner inside cancer cells, which in turn chelates Cu to produce extra H_2_O_2_. This process occurs predominantly in cancer environments, as the level of Cu is much higher than that in normal tissues. The added H_2_O_2_ can be subsequently decomposed into ^•^OH via the Fenton reaction by multiple Fe clusters in NH_2_‐MIL‐101(Fe) to allow for cancer‐specific cytotoxicity. Our CDT formulation promoted anticancer efficacy when combined with NH_2_‐MIL‐101(Fe) loaded with the anticancer drug CPT‐11. Our findings revealed that the combined formulation exhibited the highest anticancer effect in tumor‐bearing mice due to the synergistic effect of ^•^OH generation and CPT‐11 exposure, which was boosted by efficient intracellular uptake and sustained release of compounds. Therefore, NH_2_‐MIL‐101(Fe)‐based formulations represent a promising strategy for effective anticancer treatment.

## AUTHOR CONTRIBUTIONS

**Han Bi Ji:** Conceptualization (equal); data curation (equal); formal analysis (equal); investigation (equal); methodology (equal); validation (equal); writing – original draft (equal). **Cho Rim Kim:** Formal analysis (supporting). **Chang Hee Min:** Formal analysis (supporting). **Jae Hoon Han:** Methodology (supporting). **Se‐Na Kim:** Formal analysis (supporting). **Cheol Lee:** Data curation (equal); investigation (supporting); methodology (supporting); validation (supporting). **Young Bin Choy:** Conceptualization (lead); formal analysis (lead); funding acquisition (lead); investigation (lead); methodology (lead); project administration (lead); supervision (lead); validation (lead); writing – original draft (lead); writing – review and editing (lead).

## CONFLICT OF INTEREST

Han Bi Ji and Young Bin Choy are listed as inventors on the pending patents (KR 10‐2022‐0159628) filed by SNU R&DB for the formulation to reactive oxygen species described in this article.

## Supporting information


**Figure S1:** In vitro profiles of ^•^OH generation in the TMB‐containing medium of pH 5.5 PBS without Cu for (a) NH_2_‐MIL‐101(Fe)/d‐pen, (b) NH_2_‐MIL‐101(Fe) and (c) d‐pen solution. With a colorimetric probe, TMB, the content of ^•^OH was measured spectrophotometrically at 652 nm. Without the presence of Cu, there was almost no absorbance, indicating no production of ^•^OH.
**Figure S2:** SEM image of NH_2_‐MIL‐101(Fe)/calcein. The particle size and morphology did not change much after calcein loading. The scale bar is 1 μm.
**Figure S3:** In vitro release profiles of calcein from the NH_2_‐MIL‐101(Fe)/calcein. During a 1‐h cell exposure, more than 90% calcein was still entrapped in the NH_2_‐MIL‐101(Fe)/calcein, and the freed calcein would not be taken into the cells [1]. Error bars represent the standard deviation (*n* = 3).
**Figure S4:** Cytotoxicity test results with the NH_2_‐MIL‐101(Fe)/d‐pen using L929 cells. Error bars represent the standard deviation (*n* = 6).
**Figure S5:** In vitro degradation profiles of NH_2_‐MIL‐101(Fe). Error bars represent the standard deviation (*n* = 3). Almost all NH_2_‐MIL‐101(Fe) was degraded in 7 days. To assess the degradation profile, the amount of a ligand (2‐aminoterephthalic acid [2‐ATA]) freed by degradation of the NH_2_‐MIL‐101(Fe) was measured. Thus, 5 mg NH_2_‐MIL‐101(Fe) was dispersed in 1 ml PBS at pH 5.5 and 7.4 in a dialysis bag (3.5 kDa MWCO; SnakeSkin Dialysis Tubing, Thermo Fisher Scientific), respectively, and immersed in 4 ml of the same medium. The prepared sample was incubated at 37°C with stirring at 100 rpm, and at scheduled intervals, 2 ml of the supernatant was collected and replaced with an equal volume of the same fresh medium. For each collected medium, the 2‐ATA concentration was analyzed using HPLC/MS (Agilent 6120 Quadrupole LCMS Systems; Agilent Technologies, Santa Clara, CA). The chromatographic separation was performed using a Diamonsil C18 column (4.6 × 150 mm, 5 μm pore, Dikma, Lake Forest, CA) with a mobile phase pumped at a rate of 0.5 ml/min. The mobile phase was prepared by mixing MeOH and 10 mM ammonium bicarbonate (pH 8) (50:50, v/v). The sample injection volume was 10 μl, and the UV absorbance and selected ion monitoring (SIM) ion of the 2‐ATA were 420 nm and 180 m/z, respectively [2]. The degradation percentage was calculated by the following equation: Degradation (%) = Amount of freed 2‐ATA/Amount of 2‐ATA in the initially added NH_2_‐MIL‐101(Fe) × 100.

## Data Availability

The data that support the findings of this study are available from the corresponding author upon reasonable request.
