# Supplementary material for: Fe‐containing metal–organic framework with D‐penicillamine for cancer‐specific hydrogen peroxide generation and enhanced chemodynamic therapy
Source: Bioeng Transl Med. 2023 Feb 1;8(3):e10477. doi: 10.1002/btm2.10477 (PMC10189484; doi:10.1002/btm2.10477)
Supplement: Supplementary file 1 — Figure S1: In vitro profiles of •OH generation in the TMB‐containing medium of pH 5.5 PBS without Cu for (a) NH2‐MIL‐101(Fe)/d‐pen, (b) NH2‐MIL‐101(Fe) and (c) d‐pen solution. With a colorimetric probe, TMB, the content of •OH was measured spectrophotometrically at 652 nm. Without the presence of Cu, there was almost no absorbance, indicating no production of •OH. Figure S2: SEM image of NH2‐MIL‐101(Fe)/calcein. The particle size and morphology did not change much after calcein loading. The scale bar is 1 μm. Figure S3: In vitro release profiles of calcein from the NH2‐MIL‐101(Fe)/calcein. During a 1‐h cell exposure, more than 90% calcein was still entrapped in the NH2‐MIL‐101(Fe)/calcein, and the freed calcein would not be taken into the cells [1]. Error bars represent the standard deviation (n = 3). Figure S4: Cytotoxicity test results with the NH2‐MIL‐101(Fe)/d‐pen using L929 cells. Error bars represent the standard deviation (n = 6). Figure S5: In vitro degradation profiles of NH2‐MIL‐101(Fe). Error bars represent the standard deviation (n = 3). Almost all NH2‐MIL‐101(Fe) was degraded in 7 days. To assess the degradation profile, the amount of a ligand (2‐aminoterephthalic acid [2‐ATA]) freed by degradation of the NH2‐MIL‐101(Fe) was measured. Thus, 5 mg NH2‐MIL‐101(Fe) was dispersed in 1 ml PBS at pH 5.5 and 7.4 in a dialysis bag (3.5 kDa MWCO; SnakeSkin Dialysis Tubing, Thermo Fisher Scientific), respectively, and immersed in 4 ml of the same medium. The prepared sample was incubated at 37°C with stirring at 100 rpm, and at scheduled intervals, 2 ml of the supernatant was collected and replaced with an equal volume of the same fresh medium. For each collected medium, the 2‐ATA concentration was analyzed using HPLC/MS (Agilent 6120 Quadrupole LCMS Systems; Agilent Technologies, Santa Clara, CA). The chromatographic separation was performed using a Diamonsil C18 column (4.6 × 150 mm, 5 μm pore, Dikma, Lake Forest, CA) with a mobile phase pumped at a rate of 0.5 [file BTM2-8-e10477-s001.docx]

Supporting Information

**Fe-containing Metal-organic Framework with D-penicillamine for Cancer-specific Hydrogen Peroxide Generation and Enhanced Chemodynamic Therapy**

*Han Bi Ji, Cho Rim Kim, Chang Hee Min, Se-Na Kim, Jae Hoon Han,*

*Cheol Lee and Young Bin Choy*


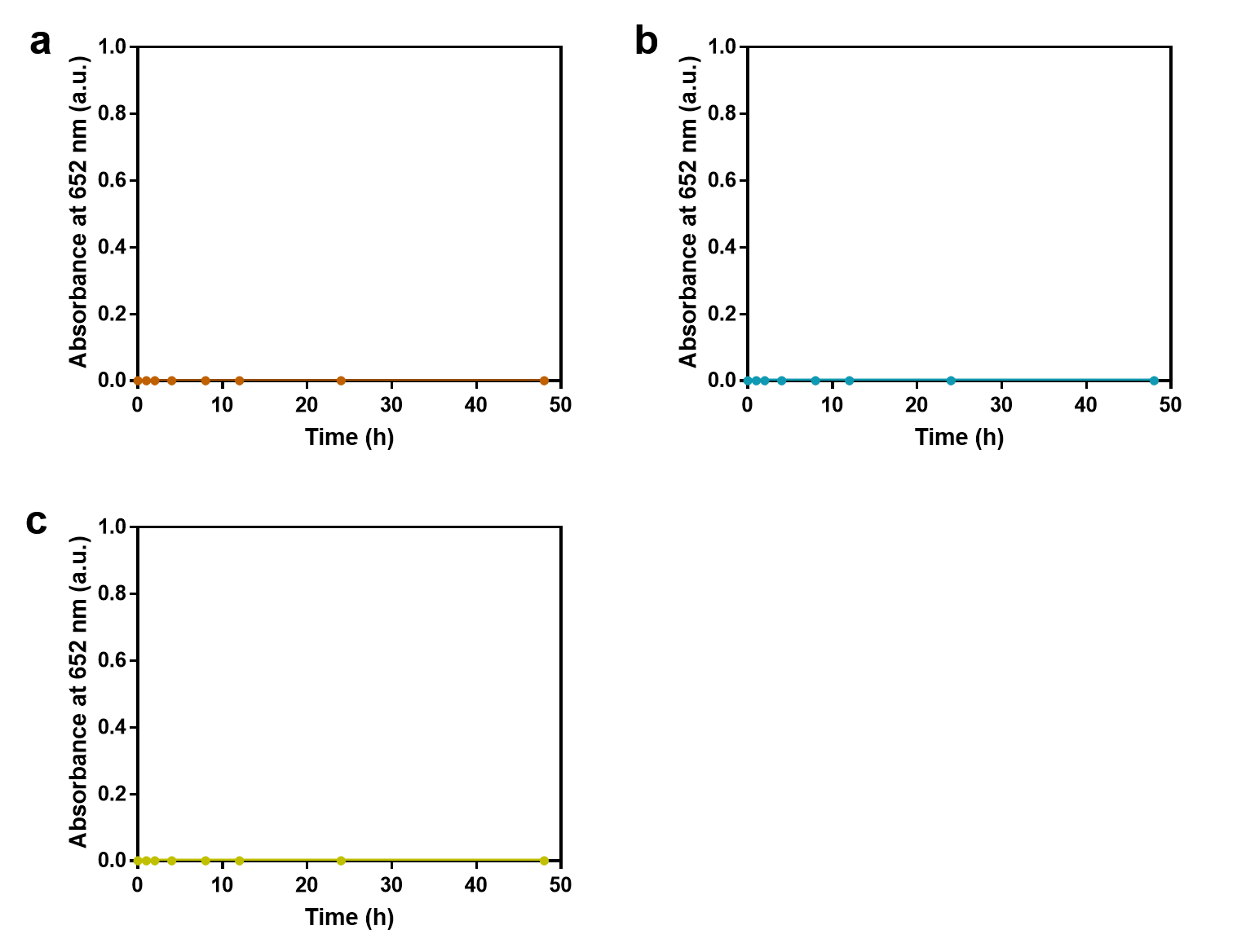


**Figure S1**. *In vitro* profiles of ^•^OH generation in the TMB-containing medium of pH 5.5 PBS without Cu for a) NH_2_-MIL-101(Fe)/D-pen, b) NH_2_-MIL-101(Fe) and c) D-pen solution. With a colorimetric probe, TMB, the content of ^•^OH was measured spectrophotometrically at 652 nm. Without the presence of Cu, there was almost no absorbance, indicating no production of ^•^OH.

**
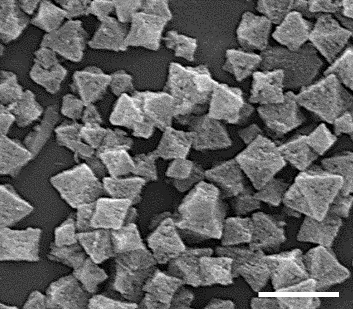
**

**Figure S2**. SEM image of NH_2_-MIL-101(Fe)/calcein. The particle size and morphology did not change much after calcein loading. The scale bar is 1 μm.


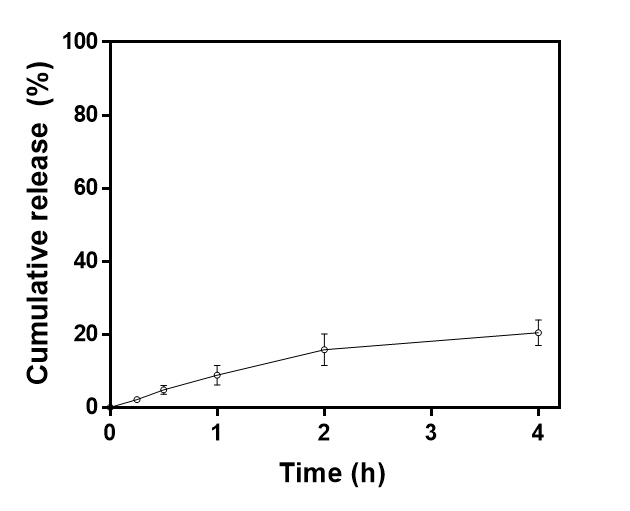
**Figure S3**. *In vitro* release profiles of calcein from the NH_2_-MIL-101(Fe)/calcein. During a 1-h cell exposure, more than 90% calcein was still entrapped in the NH_2_-MIL-101(Fe)/calcein, and the freed calcein would not be taken into the cells [1]. Error bars represent the standard deviation (n = 3).

**
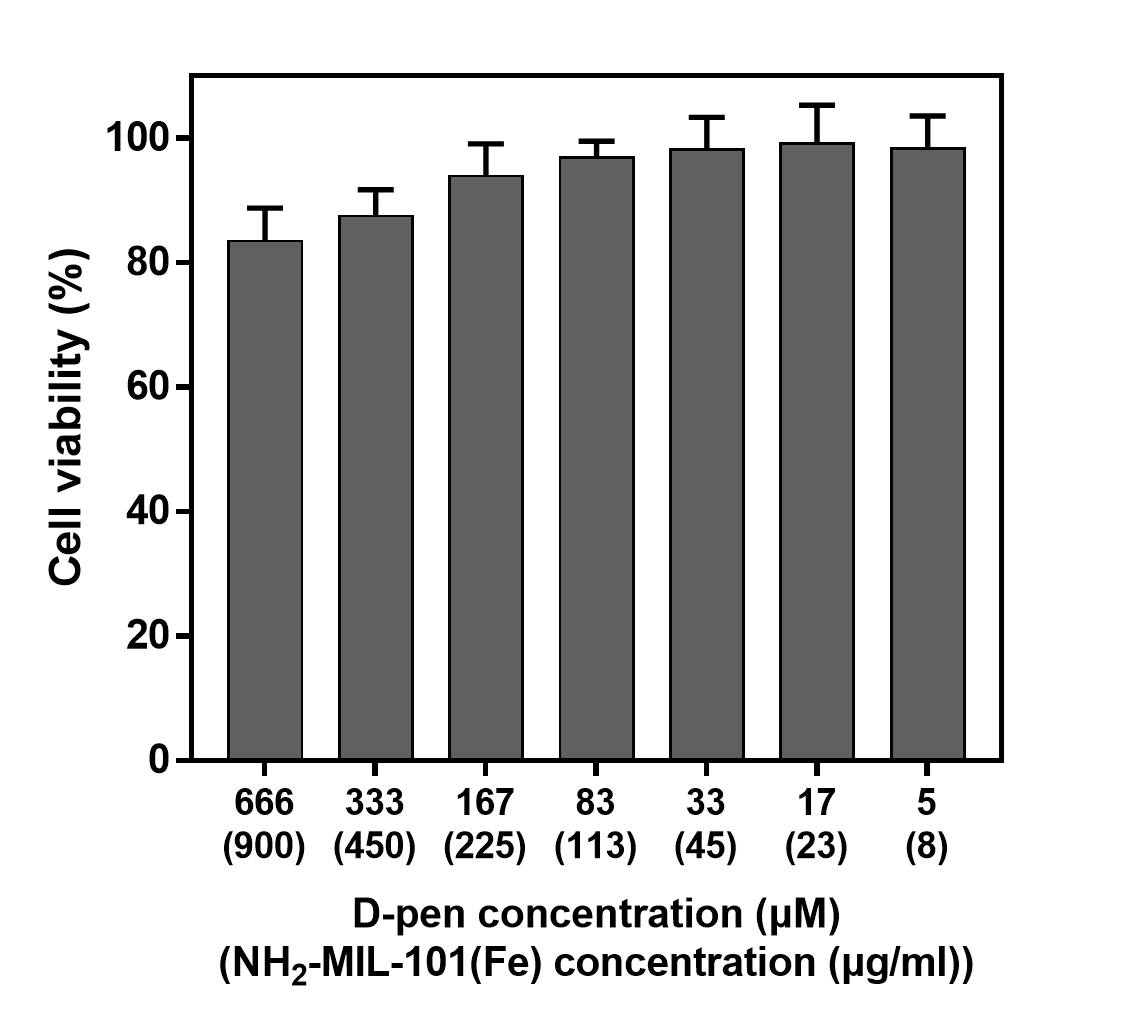
Figure S4.** Cytotoxicity test results with the NH_2_-MIL-101(Fe)/D-pen using L929 cells. Error bars represent the standard deviation (n = 6).

**
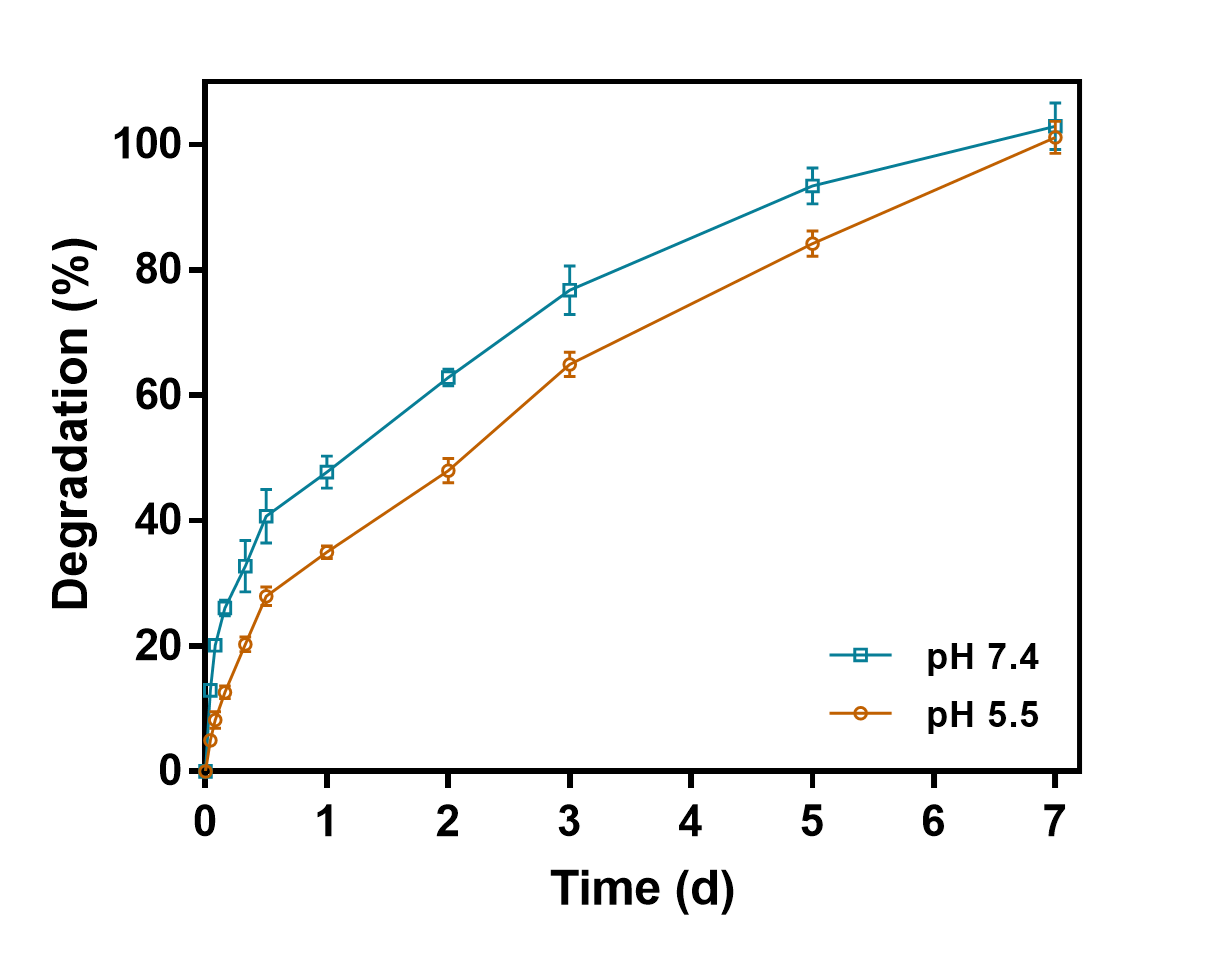
Figure S5.** *In vitro* degradation profiles of NH_2_-MIL-101(Fe). Error bars represent the standard deviation (n = 3). Almost all NH_2_-MIL-101(Fe) was degraded in 7 days. To assess the degradation profile, the amount of a ligand (2-aminoterephthalic acid (2-ATA)) freed by degradation of the NH_2_-MIL-101(Fe) was measured. Thus, 5 mg NH_2_-MIL-101(Fe) was dispersed in 1 ml PBS at pH 5.5 and 7.4 in a dialysis bag (3.5 kDa MWCO; SnakeSkin Dialysis Tubing, Thermo Fisher Scientific), respectively, and immersed in 4 ml of the same medium. The prepared sample was incubated at 37°C with stirring at 100 rpm, and at scheduled intervals, 2 ml of the supernatant was collected and replaced with an equal volume of the same fresh medium. For each collected medium, the 2-ATA concentration was analyzed using HPLC/MS (Agilent 6120 Quadrupole LCMS Systems; Agilent Technologies, Santa Clara, CA). The chromatographic separation was performed using a Diamonsil C18 column (4.6 × 150 mm, 5 μm-pore, Dikma, Lake Forest, CA) with a mobile phase pumped at a rate of 0.5 ml/min. The mobile phase was prepared by mixing MeOH and 10 mM ammonium bicarbonate (pH 8) (50:50, v/v). The sample injection volume was 10 μl, and the UV absorbance and selected ion monitoring (SIM) ion of the 2-ATA were 420 nm and 180 m/z, respectively [2]. The degradation percentage was calculated by the following equation: Degradation (%) = Amount of freed 2-ATA /Amount of 2-ATA in the initially added NH_2_-MIL-101(Fe) × 100.

**References**

1. Markopoulou P, Panagiotou N, Li A, et al. Identifying Differing Intracellular Cargo Release Mechanisms by Monitoring In Vitro Drug Delivery from MOFs in Real Time, *Cell Rep Phy Sci.* 2020;11:100254.
2. Munshi P, Pillai SM, Rao GSS, Single wavelength check isocratic reverse phase HPLC method for fast and direct estimation of alkyl aromatic oxidation products, *Anal Methods* 2020;2:382.
